# Expression of Vesicular Glutamate Transporters VGLUT1 and VGLUT2 in the Rat Dental Pulp and Trigeminal Ganglion following Inflammation

**DOI:** 10.1371/journal.pone.0109723

**Published:** 2014-10-07

**Authors:** Eun Sun Yang, Myoung Uk Jin, Jae Hyun Hong, Yun Sook Kim, So Young Choi, Tae Heon Kim, Yi Sul Cho, Yong Chul Bae

**Affiliations:** Department of Anatomy and Neurobiology, School of Dentistry, Kyungpook National University, Daegu, Korea; The Hebrew University Medical School, Israel

## Abstract

**Background:**

There is increasing evidence that peripheral glutamate signaling mechanism is involved in the nociceptive transmission during pathological conditions. However, little is known about the glutamate signaling mechanism and related specific type of vesicular glutamate transporter (VGLUT) in the dental pulp following inflammation. To address this issue, we investigated expression and protein levels of VGLUT1 and VGLUT2 in the dental pulp and trigeminal ganglion (TG) following complete Freund’s adjuvant (CFA) application to the rat dental pulp by light microscopic immunohistochemistry and Western blot analysis.

**Results:**

The density of VGLUT2− immunopositive (+) axons in the dental pulp and the number of VGLUT2+ soma in the TG increased significantly in the CFA-treated group, compared to control group. The protein levels of VGLUT2 in the dental pulp and TG were also significantly higher in the CFA-treated group than control group by Western blot analysis. The density of VGLUT1+ axons in the dental pulp and soma in the TG remained unchanged in the CFA-treated group.

**Conclusions:**

These findings suggest that glutamate signaling that is mediated by VGLUT2 in the pulpal axons may be enhanced in the inflamed dental pulp, which may contribute to pulpal axon sensitization leading to hyperalgesia following inflammation.

## Introduction

There is increasing evidence that glutamate, a major excitatory neurotransmitter in the central nervous system, also plays an essential role in the transmission of pain signals by the peripheral nervous system under normal and pathological conditions. For example, application of glutamate to peripheral tissues produces acute nociception or mechanical and thermal hyperalgesia [1]–[3]. In addition, experimental inflammation or electrical stimulation of peripheral nerve causes increase in glutamate release into peripheral tissues [4]–[6].

The vesicular glutamate transporter (VGLUT) is involved in the active loading of glutamate into synaptic vesicles before exocytotic release [7], and plays a crucial role in glutamate signaling [8]. Of the three isoforms of VGLUT, VGLUT1 and VGLUT2 are expressed by largely functionally-distinct populations of primary sensory neurons: i.e., VGLUT2 is expressed primarily by nociceptive neurons whereas VGLUT1 is expressed primarily by Aβ mechanoreceptive neurons [8]–[10]. Recently, behavioral analysis of mice with a genetic deletion of VGLUT2 in dorsal root ganglion (DRG) neurons supported the notion that VGLUT2 plays a crucial role in acute and inflammatory pain [11]–[14].

The dental pulp is richly innervated by nociceptive Aδ and C fibers [15], [16] and is thus, frequently used as a model system to study peripheral pain mechanism. Recent studies reported that the dental pulp is also innervated by a few Aβ low threshold mechanoreceptive (LTM) fibers, which may be involved in the nociception rather than in mechanosensation [17], [18]. Recently, we reported expression of VGLUT1 as well as VGLUT2 in the axons of the normal human dental pulp and that the VGLUT1 is expressed in larger number of pulpal axons than axons expressing VGLUT2, suggesting larger role of VGLUT1 than VGLUT2 in the transduction of acute nociception in the dental pulp [19]. Previous many studies have focused on the central signaling mechanism for pulpal inflammatory pain, which is dependent on glutamate. For example, application of the inflammatory irritant mustard oil to the dental pulp induces central sensitization in medullary dorsal horn, mediated by glutamate [5], [20], [21]. However, the peripheral signaling mechanism for pulpal inflammatory pain, and involvement of specific type of VGLUT in it, is largely unknown.

To address this gap in knowledge, we investigated the expression of VGLUT1 and VGLUT2 in the rat dental pulp and trigeminal ganglion (TG) in a model of dental pulp inflammation, induced by complete Freund’s adjuvant (CFA), using light microscopic immunohistochemistry, quantitative analysis of VGLUT1− and VGLUT2− immunopositive axons and somata, and Western blot analysis.

## Materials and Methods

All animal procedures were performed according to the National Institutes of Health guidelines and were approved by the Kyungpook National University Intramural Animal Care and Use Committee (permit number: KNU 2011-44). Experiments were designed to minimize the number of animals used and their suffering. Sixteen male Sprague-Dawley rats weighing 300–320 g were used for this study, including 6 for light microscopic immunohistochemistry and 10 for Western blot analysis.

### Tooth pulp inflammation model

Rats were anesthetized with sodium pentobarbital (40 mg/kg, i.p.) and the occlusal enamel and dentin of the right maxillary 1^st^ (M1) and 2^nd^ (M2) molars were filed off to just before exposing the pulp using a low-speed dental drill with a round bur under water-cooling. A small piece of tissue paper soaked in 50% CFA solution in saline was applied to the exposed dentinal surfaces for 5 minutes. Then, the dentinal surfaces were sealed with dental cement.

### Light microscopic immunohistochemistry

For immunofluorescence, on day 1 (CFA 1-day) or day 3 (CFA 3-day) following CFA application, rats were deeply anesthetized with sodium pentobarbital (80 mg/kg, i.p.), transcardially perfused with heparinized normal saline, followed by freshly-prepared fixative containing 4% paraformaldehyde in 0.1 M phosphate buffer (PB, pH 7.4). The dental pulps and TGs on the right side (experimental group) and those of the left side (control group) were carefully removed, postfixed in the same fixative for 2 hours, and cryoprotected in 30% sucrose in PB overnight at 4°C. The next day, 30-µm thick sections were cut on a freezing microtome and treated with 50% ethanol for 30 minutes and with 10% normal donkey serum (NDS, Jackson ImmunoResearch, West Groove, PA) for 30 minutes. The sections were incubated overnight in VGLUT1 or VGLUT2 antibodies in combination with protein gene product 9.5 (PGP 9.5) antibody, a marker for neuron, or with CD64 antibody, a marker for inflammatory cells [22] to confirm induction of inflammation. The primary antibodies used are as follows: guinea pig anti-VGLUT1 (#135304, 1∶2,000, Synaptic Systems, Gottingen, Germany), rabbit anti-VGLUT2 (#135402, 1∶2,000, Synaptic Systems, Gottingen, Germany), guinea pig anti-VGLUT2 (#VGLUT2-GP-Af670, 1∶1,000, Frontier Institute Co. Ltd, Hokkaido, Japan), mouse anti-PGP 9.5 (#YM8104, 1∶5,000, Accurate Chemical and Scientific Corp., Westbury, NY) and goat anti-CD64 (sc-7642, 1:500, Santa Cruz Biotechnology, CA). On the next day, the sections were washed with PB and incubated for 3 hours with a Cy3-conjugated-donkey anti-guinea pig or anti-rabbit antibody and a fluorescein isothiocyanate-conjugated-donkey anti-mouse or anti-goat antibody (1∶200, in PB, Jackson ImmunoResearch). The sections were then mounted on slides and coverslipped with Vectashield (Vector Laboratories, Burlingame, CA), and micrographs were obtained with an Exi digital camera (Q-Imaging Inc., Surrey, CA), attached to a Zeiss Axioplan 2 fluorescence microscope (Carl Zeiss Inc., Jena, Germany). To control for the specificity of the antibodies, sections were processed as described above, except that primary or secondary antibodies were omitted: Omission of primary or secondary antibodies eliminated specific staining. Preadsorption controls with blocking peptides for VGLUT1 (15 µg/ml; #135-3P, Synaptic Systems) and VGLUT2 (10 µg/ml; #G07B-VGLUT2-AG, Frontier Science) also completely abolished the respective staining.

### Quantitative analysis of immunolabeled sections

To quantify the density of the VGLUT1+ and VGLUT2+ axons and somata in the dental pulp and TG, respectively, a total of 24–30 images from 4–5 tissue sections of each of 3 dental pulps in each of CFA 1-day and CFA 3-day rats and a total of 48 images from 4 tissue sections of each side of 3 TGs in each side of CFA 1-day and CFA 3-day rats were collected; quantitative analysis of VGLUT2+ axons was obtained from sections immunostained with rabbit anti-VGLUT2 antibody (Synaptic Systems). The images were captured by a 40× objective (409.64×312.05 µm, 1360×1036 pixels) in the pulp horn area of coronal portion of the dental pulp and 20× objective (857.14×652.94 µm, 1360×1036 pixels) in the maxillary area of the TG which contains many VGLUT1+ or VGLUT2+ axons and many PGP 9.5+ axons, and many VGLUT1+ or VGLUT2+ soma, respectively. The threshold level for defining axons as immunopositive was determined at 100–120 gray levels in images with 256 gray levels using Image J software (NIH, Bethesda, MD). In the TG, only somata with clearly visible nuclei were counted. The area fraction of VGLUT1+ and VGLUT2+ axons in the dental pulp, and the count fraction of VGLUT1+ and VGLUT2+ somata of all somata in the TG were analyzed; the differences in these parameters were studied with the paired Student’s *t*-test, significance was set at p<0.01.

### Western blot

Perfusion of rats with saline and removal of dental pulps and TGs were done in the same way as for immunohistochemistry (perfusion with fixative was excluded). All chemicals, unless stated otherwise, were purchased from Sigma-Aldrich (St. Louis, MO). The samples were homogenized in extraction buffer (20 mM Tris-HCl pH 7.4, 5 mM EDTA, 140 mM NaCl, 1% Triton X-100, 1 mM Na_3_VO_4_, 1 mM PMSF, 10 mM NaF, and 1 µg/ml aprotinin) at 4°C. The extracts were centrifuged at 12,000×g for 20 minutes at 4°C. Proteins in supernatant were measured with Bio-Rad Protein Assay kit (Bio-Rad, Hercules, CA), and denatured at 95°C for 5 minutes with 5×SDS-loading buffer. Proteins were separated by electrophoresis on SDS-PAGE gel, and transferred to Immobilon-P membranes (EMD Millipore, Billerica, MA). The membranes were blocked with blocking solution (1×TBS, 5% nonfat milk, 0.02% NaN_3_) for 2 hours and incubated overnight at 4°C with primary antibodies: Mouse anti-β-actin (#sc81178, 1∶2,000, Santa Cruz Biotechnology, CA), VGLUT1 (Synaptic Systems) and VGLUT2 (Synaptic Systems) antisera were used at the same dilutions as for immunohistochemistry. After incubation, the membranes were washed with TBS and incubated with goat anti-guinea pig IgG (#sc2438, 1∶2,000, Santa Cruz Biotechnology), goat anti-rabbit IgG (#sc2004, 1∶2,000, Santa Cruz Biotechnology), or goat anti-mouse IgG (#sc2005, 1∶2,000, Santa Cruz Biotechnology) for 1 hour at room temperature. For visualization, the membranes were treated with ECL solution (EMD Millipore), according to the manufacturer’s instructions, and exposed on autoradiography film (Agfa, Mortsel, Belgium). Paired Student’s *t*-test was used to compare mean densities, significance was set at p<0.01.

## Results

### VGLUT1 and VGLUT2 expression in the dental pulp

**Figure 1:**
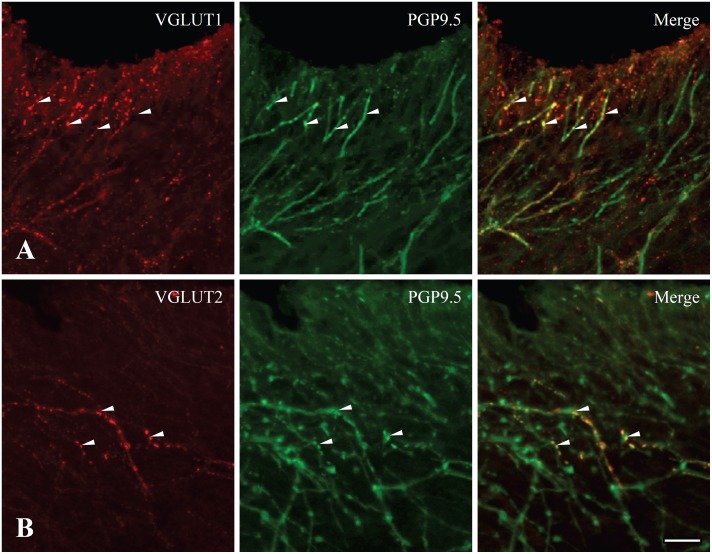
Double immunofluorescence staining for VGLUT1 (A) or VGLUT2 (B) and PGP9.5 (a marker for neuron) in coronal portion of rat dental pulp. VGLUT1- and VGLUT2- immunopositive axons costained for PGP9.5 (arrowheads) indicating VGLUT1 and VGLUT2 were expressed in pulpal axons. Scale bar = 20 µm.

**Figure 2:**
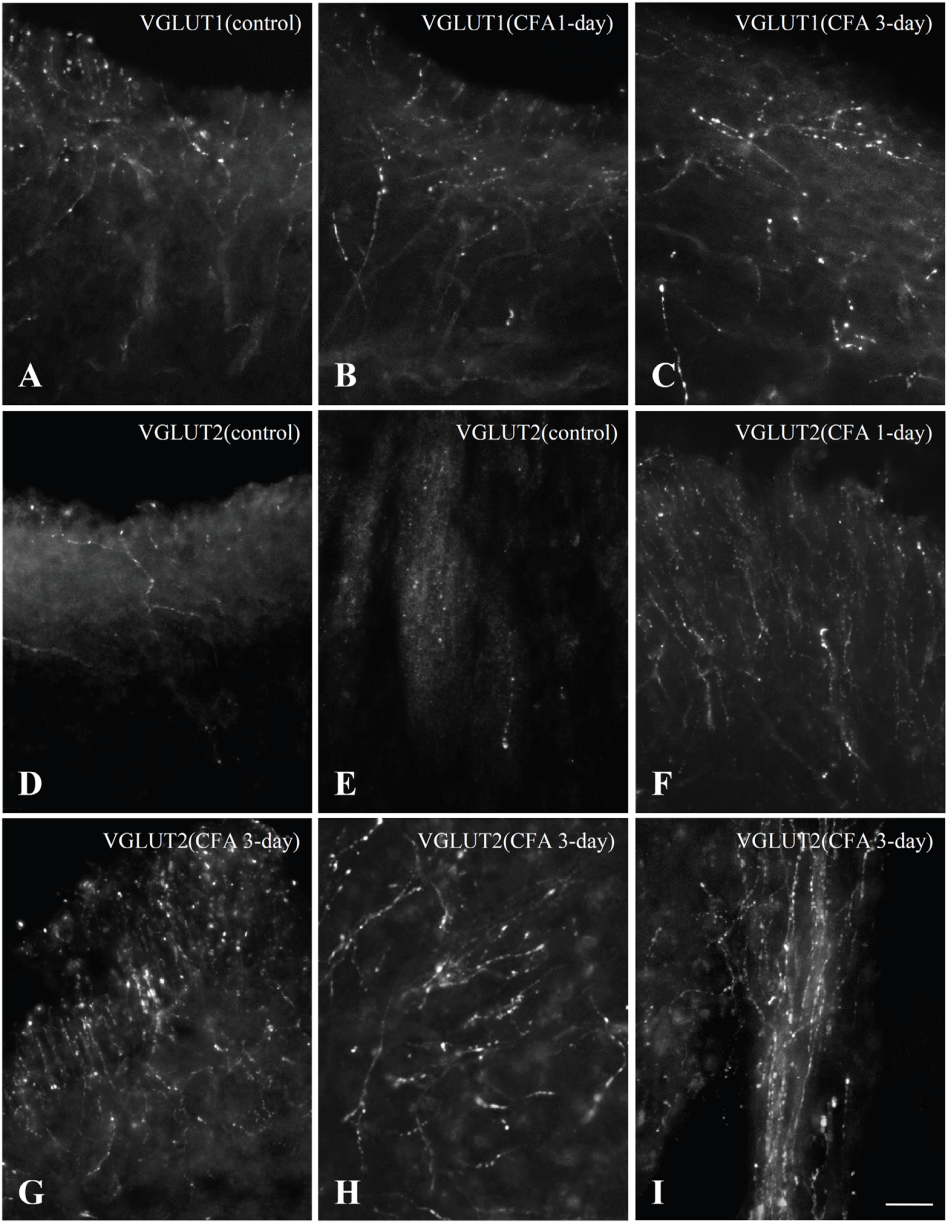
Immunofluorescent staining for VGLUT1 (A–C) and VGLUT2 (D–I) in the rat dental pulp in the control (A, D, E), CFA 1-day (B, F), and CFA 3-day (C, G–I) groups. A–C: Expression of VGLUT1 in pulpal axons in control (A) and CFA 1-day (B) and CFA 3-day (C) groups. VGLUT1 is expressed in many axons in the peripheral portion of the coronal pulp. **D–I:** Expression of VGLUT2 in pulpal axons in control (D, E), CFA 1-day (F), and CFA 3-day (G–I) groups. In the control group, VGLUT2 is expressed in a small number of axons in the peripheral portion of coronal pulp (D) and few axons in the radicular pulp (E). However, it is expressed in a large number of axons in the peripheral portion (F, G), the core of the coronal pulp (H), and in radicular pulp (I) in the CFA 1-day and CFA 3-day groups. Scale bar = 20 µm.

**Figure 3:**
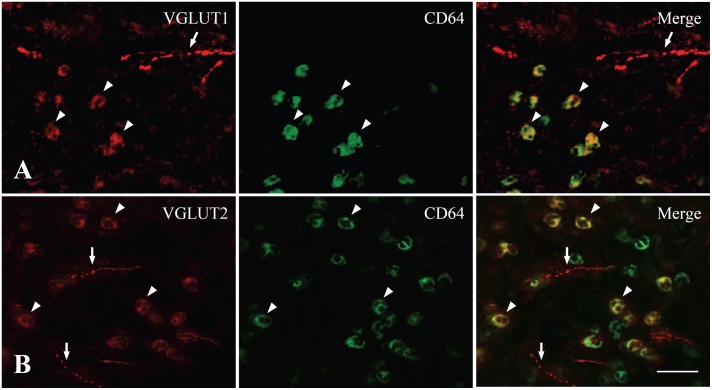
Double immunofluorescent staining for VGLUT1 and CD64 (A) and for VGLUT2 and CD64 (B) in the rat dental pulp in CFA 1-day group. VGLUT1 and VGLUT2 are expressed in many CD64+ cells (arrowhead) as well as in axons (arrow) in the inflamed dental pulp. Scale bar = 20 µm.

In the dental pulp of controls, VGLUT1 and VGLUT2 were expressed in axons (Fig. 1A, B). VGLUT1− and VGLUT2− immunopositive (+) axons were numerous in the peripheral pulp and the pulp horn and few in the core of the coronal pulp and in the radicular pulp (Fig. 2A–I). In the dental pulp of CFA-treated group, CD64+ inflammatory cells were observed, indicating induction of inflammation, and VGLUT1 and VGLUT2 were expressed in axons but also in CD64+ inflammatory cells (Fig. 3).

**Figure 4:**
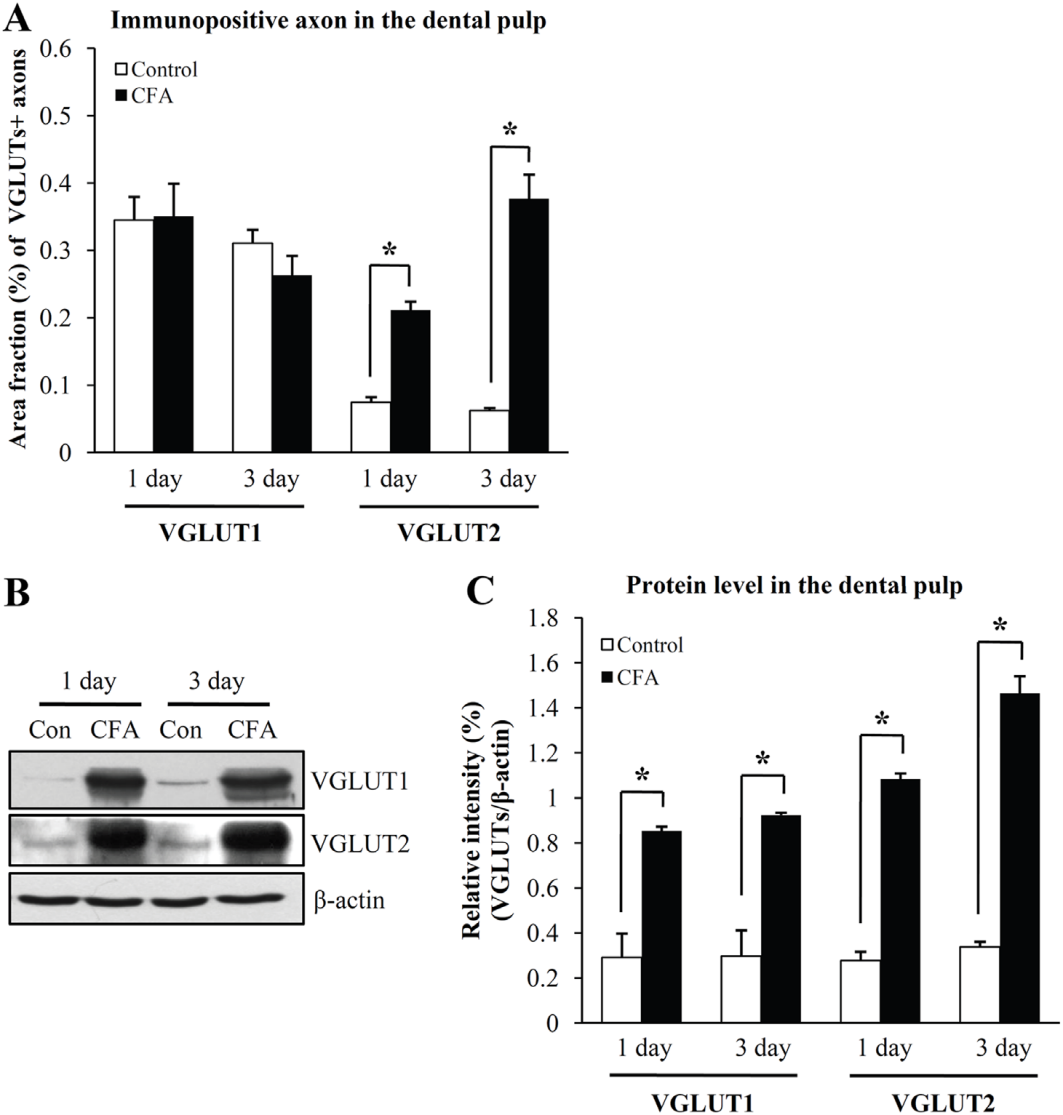
Density of VGLUT1+ and VGLUT2+ axons (A, immunofluorescence assay) and protein levels of VGLUT1 and VGLUT2 (B, C, Western blot assay) in the control, CFA 1-day and 3-day pulps. **A**: The density (area fraction) of VGLUT1+ pulpal axons is not significantly different between CFA 1-day or CFA 3-day groups and control, whereas the density of VGLUT2+ axons is significantly higher in the CFA 1-day, CFA 3-day groups than control. N = 3 animals in each group. *p<0.01. **B**: Representative images of the Western blot assay. **C**: Quantitative analysis of VGLUT1 and VGLUT2 protein in the dental pulp. Protein levels of VGLUT1 and VGLUT2 in the pulp are significantly higher in the CFA 1-day, CFA 3-day groups than control. This difference is bigger for VGLUT2 (3.9 and 4.3 fold higher in the CFA 1-day and CFA 3-day groups than control) than for VGLUT1 (2.9 and 3.1 fold higher in the CFA 1-day and CFA 3-day groups than control). N = 5 animals in each group. *p<0.01.

The density of VGLUT1+ axons was not significantly different between CFA-treated and control groups (Fig. 2A–C, 4A), whereas the density of VGLUT2+ axons was 3.0 fold higher in the CFA 1-day group and 6.3 fold higher in CFA 3-day group than control group (Figs. 2D–I, 4A). The increase in the number of VGLUT2+ axons was particularly obvious in the peripheral pulp. VGLUT2+ axons showing extensive branching, which were rarely observed in the control pulp, were frequently observed in the pulp of CFA-treated rats (Fig. 2F–H). The number of VGLUT2+ axons was also greatly increased in the radicular pulp of the CFA 3-day rats (Fig. 2I).

Protein levels of VGLUT1 and VGLUT2 in the dental pulp increased significantly in the CFA-treated groups, compared to the control group (Fig. 4B, C). This increase was much larger for VGLUT2 than for VGLUT1 (VGLUT1 and VGLUT2 levels were 2.9 and 3.9 fold higher than control, respectively, in the CFA 1-day rats, and 3.1 and 4.3 fold higher than control, respectively, in the CFA 3-day rats).

### VGLUT1 and VGLUT2 expression in the TG

**Figure 5:**
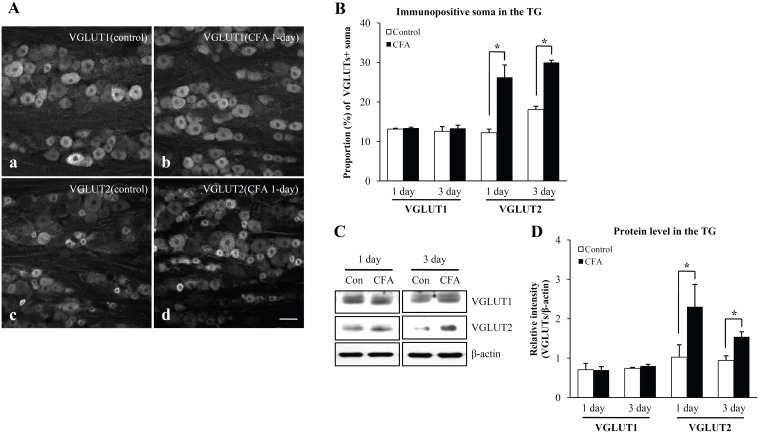
Immunofluorescent staining for VGLUT1 and VGLUT2 (A), density of VGLUT1+ and VGLUT2+ somata (B, immunofluorescence assay), and protein levels of VGLUT1 and VGLUT2 (C, D, Western blot assay) in the trigeminal ganglion. **A:** Immunofluorescent staining for VGLUT1 (a, b) and VGLUT2 (c, d) in the rat trigeminal ganglion in the control (a, c) and CFA 1-day (b, d) groups. VGLUT1 is expressed predominantly in medium- and large-sized somata (a, b), whereas VGLUT2 is expressed predominantly in small- and medium-sized somata (c, d). The number of VGLUT1+ somata of all somata is not different between control (a) and CFA 1-day group (b), whereas that of VGLUT2+ soma is significantly higher in the CFA 1-day (d) than in the control (c) groups. Scale bar = 50 µm. **B:** The density of VGLUT2+ somata (fraction of all somata) is significantly higher for the CFA 1-day, CFA 3-day groups than control, whereas the density of VGLUT1+ soma is not significantly different between CFA 1-day or CFA 3-day groups and control. N = 3 animals in each group. *p<0.01. **C:** Representative images of the Western blot assay for VGLUT1 and VGLUT2 in the rat trigeminal ganglion. **D:** Quantitative analysis of VGLUT1 and VGLUT2 protein in the trigeminal ganglion. The VGLUT2 protein levels are significantly higher in the CFA 1-day and CFA 3-day groups than for the control, whereas the VGLUT1 protein levels are not different between CFA 1-day or CFA 3-day groups and the control group. N = 5 animals in each group. *p<0.01.

In control TG, VGLUT1 was expressed mainly in medium- and large-sized somata, whereas VGLUT2 was expressed mainly in the small- and medium-sized somata (Fig. 5A). The density of VGLUT1+ somata and the level of VGLUT1 protein in the TG were not significantly different between CFA-treated group and control group. However, the density of VGLUT2+ somata and the level of VGLUT2 protein in the TG increased significantly in the CFA-treated group compared to the control group (Fig. 5B–D).

## Discussion

The main finding of this study is that the expression of VGLUT2, but not VGLUT1, increased significantly in the pulpal axons and TG somata following CFA-induced pulpal inflammation. It suggests that VGLUT2− mediated glutamate signaling by pulpal axons is enhanced following pulpal inflammation, which may contribute to the sensitization of pulpal axons during inflammation and thus to inflammatory pain.

Electrophysiological studies have shown that application of mustard oil to the dental pulp induces an increase in glutamate release and sensitization of nociceptive neurons in the medullary dorsal horn [5], [23]. In addition, mice in which VGLUT2 is ablated selectively from dorsal root ganglion (DRG) neurons, show decreased responses to acute noxious stimuli, and attenuated mechanical or thermal responses in a model of neuropathic and inflammatory pain, suggesting that VGLUT2− dependant glutamate signaling plays a crucial role in acute, neuropathic, and inflammatory pain [11]–[13], [24]. These studies also indicate that the VGLUT2− mediated glutamate signaling by the central endings of nociceptive afferents may be enhanced following inflammation, thus contributing to central sensitization and inflammatory hyperalgesia. The increase in the VGLUT2, but not VGLUT1, expression in the pulpal axons and TG somata following pulpal inflammation in our study provides a morphological evidence supporting the notion that the peripheral glutamate signaling by the pulpal axons may be also enhanced following pulpal inflammation, and that it may be mediated by VGLUT2: It was shown that expression level of VGLUT is closely related with the amount of glutamate release [25], [26]. It corroborates previous studies showing increased glutamate release in peripheral tissues following inflammation [6], [27], [28], but is at variance with a recent study showing that the number of neurons that express mRNA for VGLUT1 and VGLUT2 in the mouse DRG does not change following CFA-induced inflammation [29]. Possible explanations of this discrepancy include differences in the detection methods (immunohistochemistry vs. in situ hybridization), species (rat vs. mouse), and peripheral tissues to which CFA was applied (dental pulp vs. plantar skin) between the present and previous studies. The upregulation of the VGLUT in this study may have been also, in part, due to the pulp injury by the reduction of the dentin. Glutamate released from the pulpal nociceptive axons may act on the releasing axon itself (autocrine) or on nearby axons (paracrine) to sensitize these axons. It can also evoke the release of neuropeptides and vasoactive substances from axons [30], [31], which may contribute to the development of neurogenic inflammation [32], [33]. Recent studies showed that orofacial tissue inflammation induces excitability increase of TG neurons innervating the inflamed tissue as well as those innervating non-inflamed tissue, and that this is caused by neuronal interaction among TG neurons: Thus, TRPV1 expression is increased in the TG neurons innervating inflamed as well as non-inflamed tissues, which is caused by stimulation of the adjacent TG neurons by molecules released from the TG neurons innervating inflamed tissue [34]–[36]. In the present study, dramatic increase in VGLUT2+ neurons and VGLUT2 protein in the TG was observed though inflammation was induced in maxillary 1^st^ and 2^nd^ molar pulps, which are innervated by only a limited number of TG neurons. Considering the above mentioned reports, this might be also caused by neuronal interaction by various molecules released from the TG neurons innervating the inflamed pulps. Analysis of the VGLUT2 expression, in the present study, was performed only in the maxillary area of the TG innervating inflamed pulps. Given the cross-excitation of TG neurons following inflammation, VGLUT2 expression can be increased also in the mandibular area of the TG following inflammation of the maxillary molar pulps.

Dental pulp is primarily innervated by Aδ and C fibers, but also by many Aβ fibers [16]. Existence of pulpal Aβ fibers is also supported by many studies showing that many neurons innervating dental pulp are large- and medium-sized [37], [38], and are not depleted by neonatal capsaicin treatment [39]. In addition, many studies reported that VGLUT1 is primarily expressed in the LTM Aβ fibers whereas VGLUT2 is primarily in small-diameter nociceptive fibers [9], [40]–[42]. These reports suggest that VGLUT1 may be expressed in LTM Aβ fibers and VGLUT2 in nociceptive Aδ and C fibers in the dental pulp. Many studies suggested that pulpal LTM Aβ fibers may be involved in the nociception: Thus, all kinds of stimulations on the dental pulp result in the subjective experience of pain [15], [43]. In addition, many of the large neurons innervating dental pulp, which may have Aβ fibers, terminate in the superficial lamina of the medullary dorsal horn [44] and express peptides and neurotrophic factors which are normally associated with C fiber nociceptors [39], [45]. In the present study, VGLUT1 was expressed in many pulpal axons, which is consistent with the previous study in the human dental pulp [19]. In addition, the density of VGLUT1+ axons and soma in the dental pulp and TG, respectively, in contrast to that of VGLUT2+ axons and soma, was not significantly different between control and CFA-treated group. Given that VGLUT1 may be expressed in many pulpal LTM Aβ fibers that may be involved in nociception, it is possible to assume that VGLUT1 in the pulpal axons can be involved in the glutamate signaling related to acute pulpal pain, while VGLUT2 in pulpal axons are primarily involved in the inflammatory pain.

Recent studies showed that VGLUTs are also expressed in non-neuronal cells, suggesting the possibility of glutamate release from non-neuronal cells, including pinealocytes in the pineal gland [46], α cells in the Langerhans islets [47], and osteoclasts in bone [46]. Glutamate release is also increased in peripheral tissues during inflammation [27], [48] from nociceptive afferents, but also from non-neuronal cells, like Schwann cells [49], neutrophils [50], and macrophages [51]. VGLUT has also been shown to increase in fibroblasts and infiltrating leukocytes in inflamed tendon and muscle [52], [53]. This is in agreement with our observation of VGLUT1 and VGLUT2 expression in CD64+ inflammatory cells following inflammation. In the present study, the protein level of VGLUT1 increased in the dental pulp, though the density of VGLUT1+ axons was not changed, in the CFA-treated group than control group. This might be caused by VGLUT1 expression in the CD64+ inflammatory cells in the dental pulp following inflammation. Activation of glutamate signaling by non-neuronal cells in the inflamed pulp can play a further role in sensitization of pulpal axons and development of inflammatory hyperalgesia.
